# GWAS of canines identifies potential aging determinants

**DOI:** 10.18632/aging.203661

**Published:** 2021-10-27

**Authors:** Timothy M. Robinette, Sergiy Libert

**Affiliations:** 1 Department of Biomedical Sciences, Cornell University, Ithaca, NY 14853, USA; 2 Department of Obstetrics, Gynecology, and Reproductive Sciences, Magee-Womens Research Institute, University of Pittsburgh School of Medicine, Pittsburgh, PA 15213, USA; 3 Calico Life Sciences, South San Francisco, CA 94080, USA

**Keywords:** metabolism, GWAS, age-related phenotypes, disease, mammalian aging

Age-related metabolic changes, such as decline of respiration rate, decline of beta oxidation, and shift towards glycolysis directly increase the risk for many diseases such as obesity, type II diabetes mellitus (T2DM), and certain cancers. Moreover, these metabolic disturbances also increase susceptibility to various acute diseases indirectly, due to their negative impact on the immune system. For example, metabolism-induced decline in the function of B-cells and macrophages results in the increased rates of severe infections, auto-immune diseases, and decline in the efficiency of immunizations. These considerations suggest that identification and therapeutic targeting of key metabolic pathways could help prevent and ameliorate numerous diseases and disorders associated with aging.

Different breeds of dogs have highly diverse metabolic rates and profiles. A recently published comparative study, which analyzed different dog breeds with average lifespans varying from 6 to 22 years revealed interesting links between metabolism and longevity. Longer-lived dog breeds tend to have more uncoupled mitochondria, less electron escape, higher capacity for respiration, higher rates of amino acid catabolism, higher levels of beta-oxidation, and enhanced stress resistance [1].

The finding that longer-lived dog breeds have more uncoupled mitochondria supports the uncoupling-to-survive theory. The explanation of how uncoupled mitochondria translates into longer life remains elusive, but several published studies have supported this theory. For example, Rose et al. demonstrated that uncoupling proteins are positive modulators of human longevity [2]. In a separate study, authors showed that variations in the proton leak process contribute to obesity development or weight loss and in rats [3], and variations in proton leak could account for almost 20-30% of the resting metabolic rate.

While uncoupled mitochondria tend to be beneficial for extending lifespan, an efficient electron transport chain is favorable for longevity. Unfortunately, the efficacy of the ETC declines with advancing age, leading to a greater “electron leakage” (transfer of electrons onto unintended targets), which simultaneously increases the rate of ROS (reactive oxygen species) generation and reduces ATP production [4]. High levels of ROS have been demonstrated to be detrimental for lifespan, though the role in life determination has been repeatedly challenged [5]. It is interesting however, that long-lived dogs have less electron escape and simultaneously have a higher capacity for respiration [1]. These data suggest that genetic variants that modify stability of the ETC and mitochondrial function either aid or co-evolve with longevity. It would be interesting to test the causality of ETC-longevity association by performing longevity experiments in genetically identical animals with altered ETC mitochondrial genomes (e.g. short-lived dog breeds with mitochondria from long-lived breeds).

Cells from longer lived dogs tended to have better resistance against oxidative stress. Adaptive homeostasis in the cell depends on redox signaling, which targets NRF2, NF-κB, AMPK, uncoupling proteins, proteostasis, and mitochondrial signaling [5]. However, with age, the efficiency of oxidative stress responses decline, and excessive oxidative damage can lead to various diseases associated with aging. Potential therapeutic approaches involve upregulating the NRF2 system, utilizing suppressors of O2-, controlling the amount of free iron, or attempting to chelate free radicals with antioxidants [5]. Another approach to influence metabolic state is altering nutritional profile, such as ketogenic diets, which have been used to treat T2DM, obesity, to significantly improve global cognition, memory, and executive functions in patients at multiple stages of Alzheimer’s disease (AD) [6].

It is generally accepted that nutrient availability signaling (such as TOR signaling pathways) [7] accelerates aging while lowering such signaling extends longevity. Many nutrient sensing pathways are essential, however, and if reduced below a certain limit will lead to death. The authors determined that the long-lived dogs in general have higher rates of amino acid and fatty acid catabolism, suggesting that such metabolic adaptations might be beneficial for longer life and extended healthspan.

In addition to analysis of metabolic properties of dogs of different breeds, the authors also performed a genome-wide association study (GWAS) of average breed lifespan and identified multiple loci associated with canine longevity. One of the strong candidates for canine longevity is FGF5. This gene was shown to control the length of fur in dogs, sheep, and goats. It is possible that gene variants that impact mitochondrial uncoupling, the process mainly used for thermogenesis, and gene variants that impact length of fur, which modulates heat retention, co-evolved throughout history. Intriguingly, FGF5 gene variants are associated with blood pressure in humans in a UK Biobank (UKBB) study of around 500,000 volunteers [8], which is a major predictor of cardiovascular health and survival. Additionally, FGF5 gene variants are associated with male pattern balding, which is an age-dependent phenotype. Taken together, it is possible that FGF5 impacts mammalian aging in a more wholesome way.

Other mitochondrial and bioenergetic regulating genes that were associated with breed life expectancy were IGFBP2, MSRB3, ATP23, and TRMT5. Interestingly, in humans, each gene was found to be associated with age-related phenotypes. IGFBP2 was found to be associated with high cholesterol and triglyceride levels, osteoporosis, hypothyroidism, and Parkinson's Disease. MSRB3 was implicated in affecting basal metabolic rate, right hippocampal volume, which decreases with age, lung cancer, and diabetes. ATP23 was involved in the development of lung cancer and Alzheimer's Disease. TRMT5 impacted bone fractures, osteoarthritis, larynx/throat cancer, and diabetes. They were all associated with high blood pressure.

**Figure 1:**
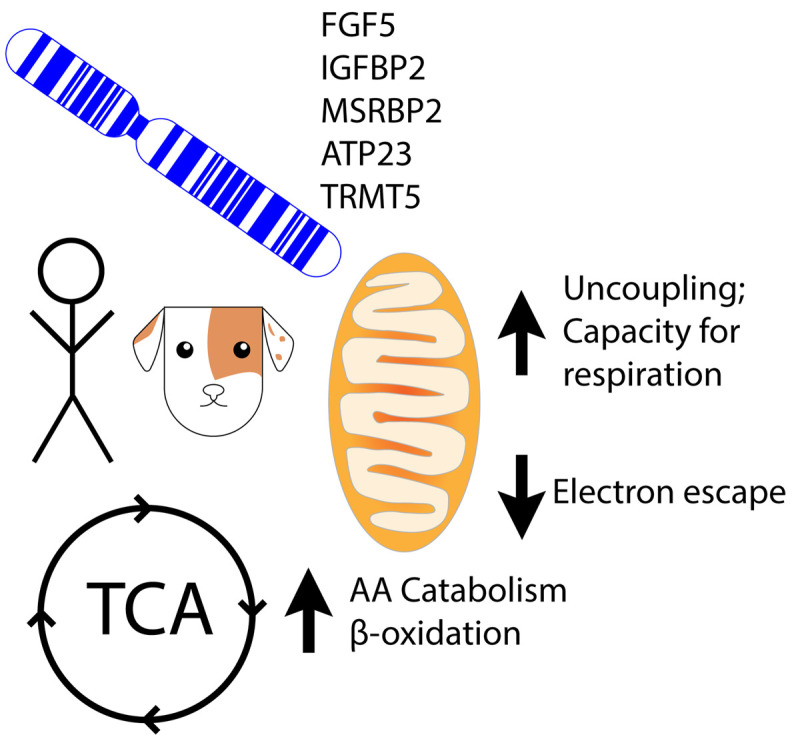
**Human and canine genes are linked to aging.** In canines, these genes potentially impact pro-longevity effects shown above.

Overall, the study suggests new genetic targets and pathways that can be used to ameliorate diseases associated with metabolic disorders and advanced age, and aging overall (Figure 1).
